# The silhouette technique: improving post-operative radiographs for planning of correction with a hexapod external fixator

**DOI:** 10.1007/s11751-017-0287-5

**Published:** 2017-05-12

**Authors:** Jonathan Wright, Shiraz A. Sabah, Shelain Patel, Gavin Spence

**Affiliations:** 1 Specialty Registrar, Great Ormond Street Hospital for Children, London, WC1N 3JH UK; 2 Consultant Paediatric Orthopaedic Surgeon, Great Ormond Street Hospital for Children, London, WC1N 3JH UK

**Keywords:** Radiograph, Deformity correction, Hexapod, Taylor spatial frame, Planning

## Abstract

Correction of deformity of a bone through use of a hexapod external fixator requires clear definition of the relationship between the bone and the frame. Achieving adequate orthogonal calibrated radiographs for this aim, with minimum X-ray exposure, can prove a challenge in the radiography suite. We describe a simple technique for obtaining adequate imaging, without the use of additional equipment. Introduction of the technique to our department has demonstrated an improvement in the adequacy of planning radiographs and a reduction in the requirement for repeat imaging.

## Introduction

The hexapod external fixator is a versatile tool, used for treatment of fractures, mal/non-unions and limb deformities in both children and adults. There are several systems available, including the Taylor Spatial Frame (Smith & Nephew, Memphis, TN), TL-Hex (Orthofix, Verona, Italy), Ortho-SUV (Ortho-SUV Ltd, St. Petersburg, Russia). The scope for correction of multiplanar deformity requires an accurate and precise definition of the starting point of the frame, in order that the desired end point is reached [1, 2]. An incorrect definition of these “mounting parameters” has been implicated in residual deformity at the end of correction, requiring adjustment of the correction programme in up to one-third of patients [3–6].

Radiographic methods to determine mounting parameters require “true” anteroposterior (AP) and lateral images, each perfectly orthogonal to the plane of the reference ring. This may be difficult to achieve in practice, resulting in multiple exposures, increased radiation dose, and even repeat visits to the radiology department if the information is not sufficient to plan the correction. Various methods have been described, involving use of rancho cubes, rods or bolts to assist alignment [2, 7, 8], use of a frame mounted spirit level [9] or construction of a guide rail in the radiology department [10] to assist in obtaining true orthogonal images.

We describe a method used in our department to reliably obtain adequate orthogonal images, which utilises the resources available in any radiology department, without requirement for adjustment or augmentation of the frame. For the purposes of this technique, we have used the Taylor Spatial Frame (TSF) as an example. We have also audited the effect on the rates of adequate radiographs over the introduction of this technique.

## Methods

### The “silhouette technique”

**Fig. 1:**
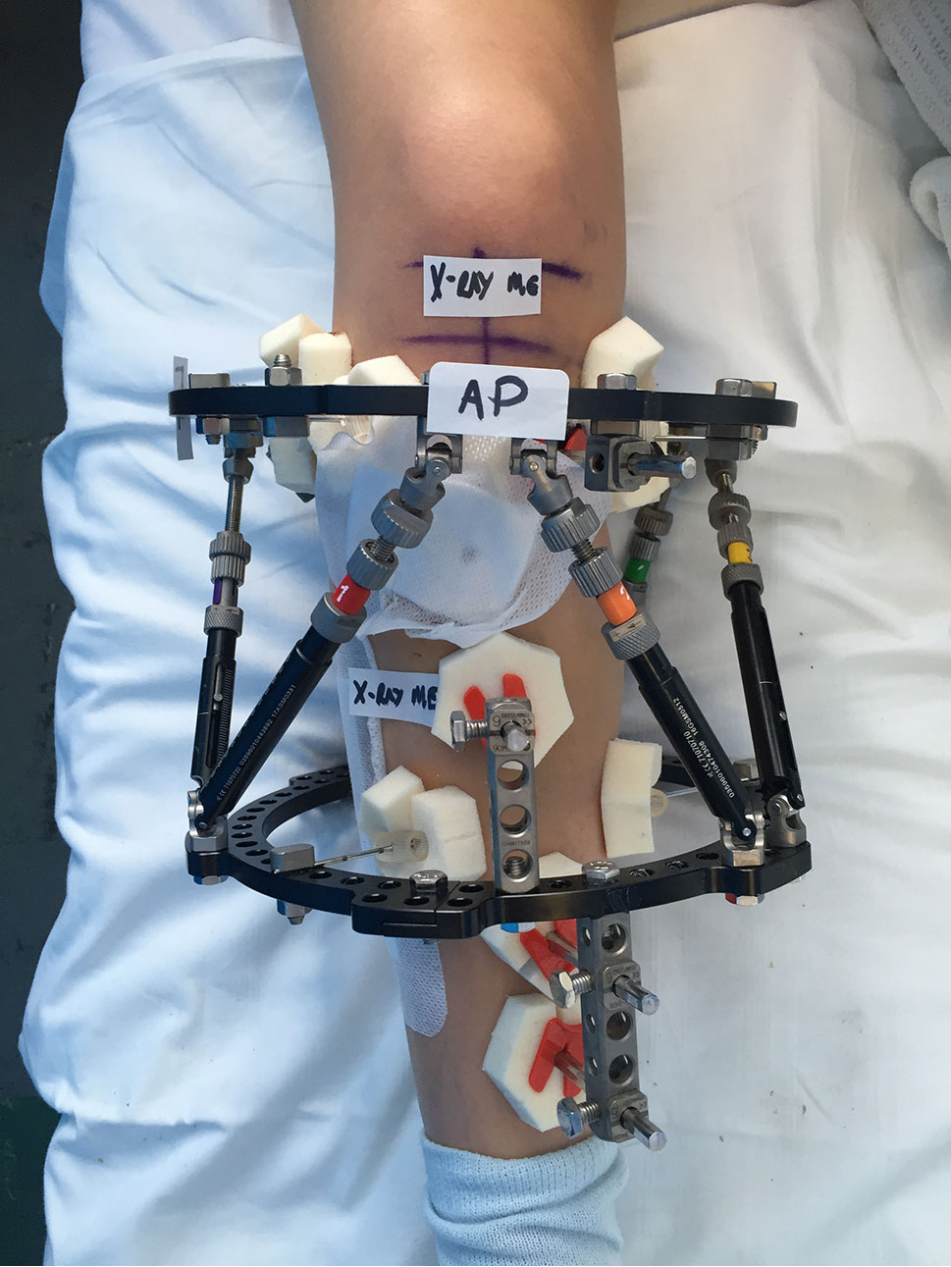
Post-operative photograph of TSF applied to right tibia. *Stickers* have been placed on the reference ring (proximal ring in this case), and the AP sticker is placed over the master tab. Further stickers denote the minimum field of view

**Fig. 2:**
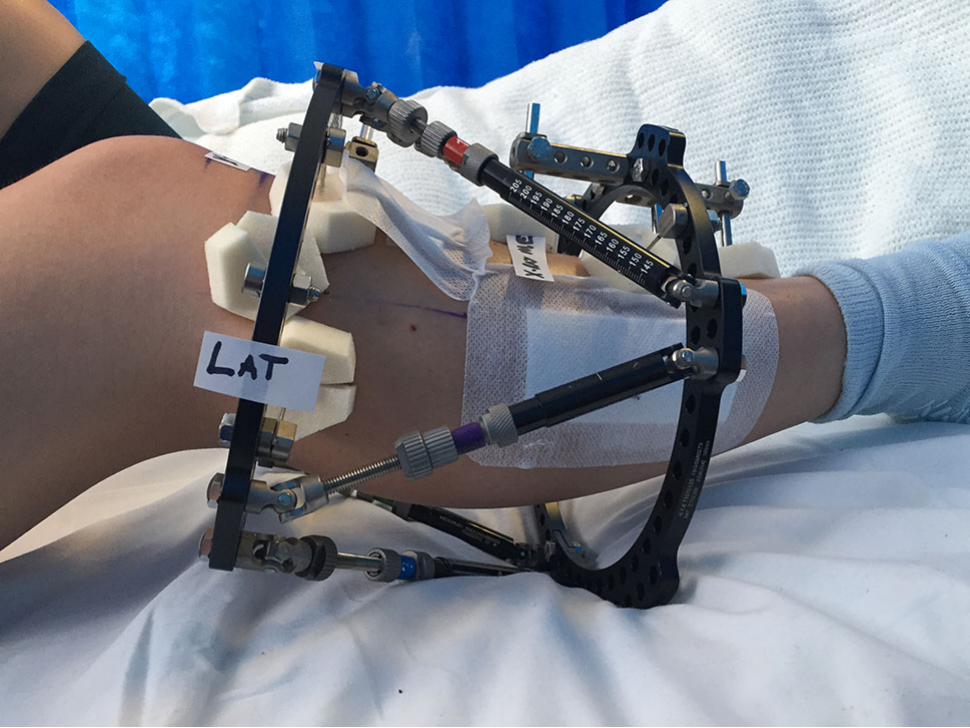
Post-operative photograph demonstrating positioning of “lateral” sticker indicating centre of X-ray beam

The silhouette technique utilises the light source and cross-hair localiser, used in most standard departmental X-ray machines. The orientation and field of view are indicated by stickers applied to the reference ring by the surgical team on the ward before post-operative imaging. These stickers indicate the centre of the reference ring (marked AP), the centre on the lateral view (90° to the AP, marked lateral) and the proximal and distal extent of the limb that must be included in the field of view (marked “X-ray me”; Figs. 1, 2).

**Fig. 3:**
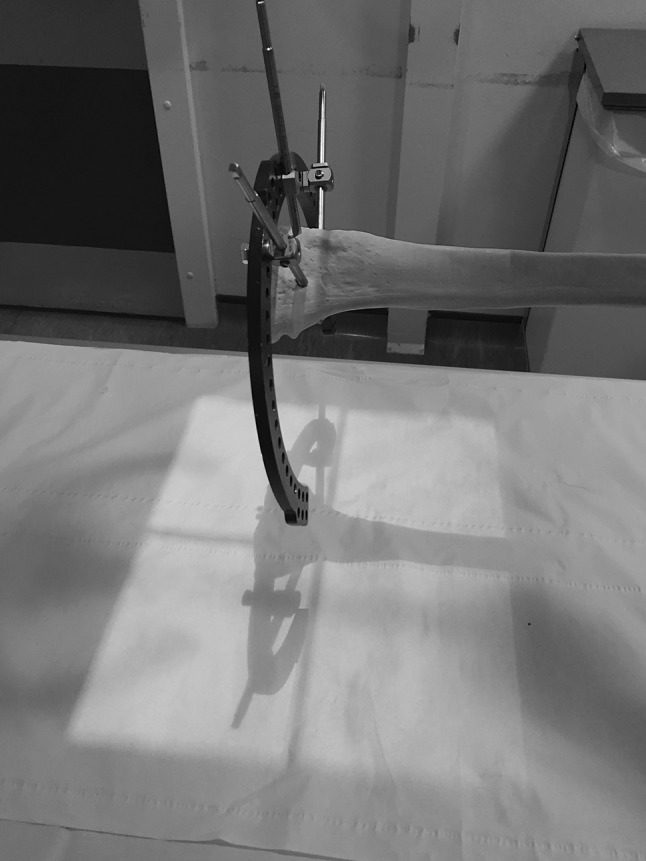
An *elliptical shadow* created by the ring can be seen in this bone model of a frame, indicating an oblique alignment

**Fig. 4:**
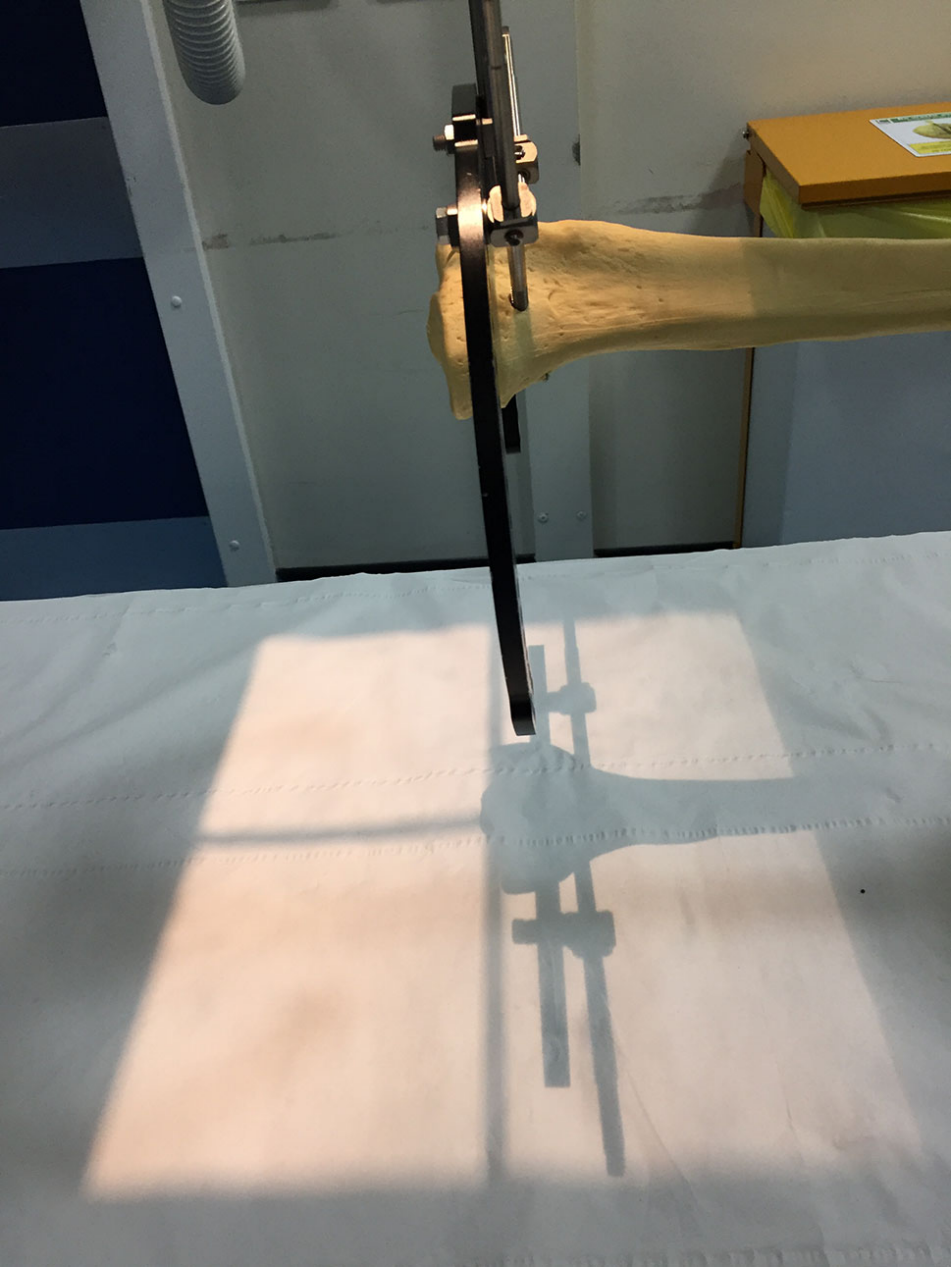
The *shadow* created by the ring is now a *single line* indicating the X-ray beam is orthogonal to the plane of the ring

The X-ray source cross-hairs are then centred on to the reference frame stickers and the light from the X-ray source used to project a shadow from the TSF onto the X-ray plate (Fig. 3). The X-ray source is then adjusted until the silhouette from the reference ring is projected as a single line, indicating an orthogonal view (Fig. 4).

**Fig. 5:**
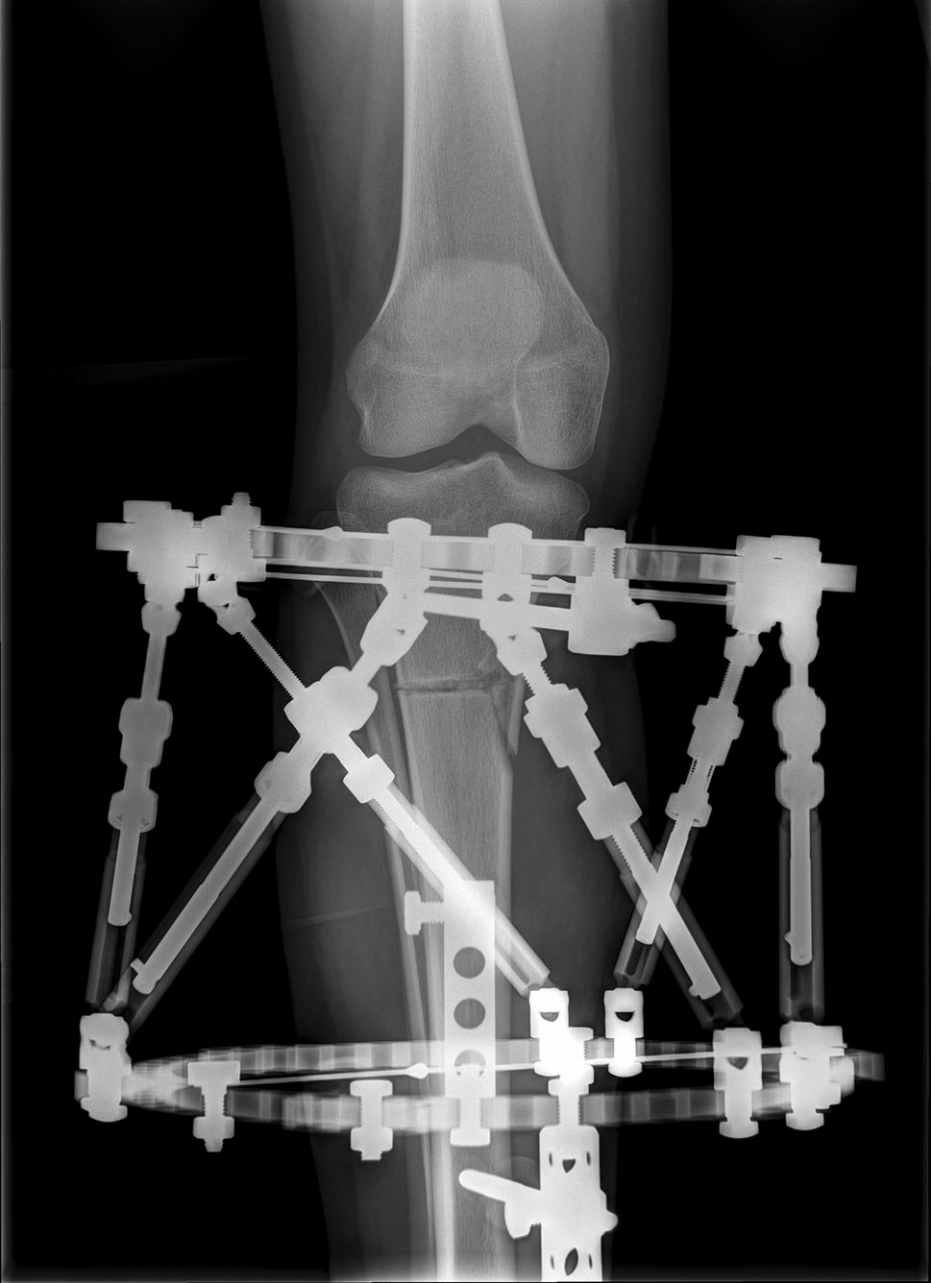
AP radiograph of the right tibia: an adequate radiograph taken orthogonal to and including the entire reference ring allowed sufficient visualisation of any deformity and the osteotomy

The field of view is then enlarged to encompass the marker ball, entire reference ring and pre-specified minimum area on a single exposure with adjacent joint(s), and entire long bones and the other elements of the frame can also be accommodated in this exposure or “stitched on” from an additional exposure (Fig. 5).

### Service evaluation: outcome of technique

The adequacy of post-operative radiographs following frame application was assessed prior to the introduction of the new technique and then for a 3-month period following this. Information was gathered on: age of patient, underlying aetiology, radiation dose, date of surgery, date of first radiograph and adequacy of radiographs. X-ray requests included the information “Post-operative images of TSF for planning. AP and lateral radiographs orthogonal to reference ring please”.

An adequate radiograph was defined as one that included the entire reference ring, with a sufficient view of the long bone to determine its axis, allowing planning on the Taylor Spatial Frame Web site to generate a prescription.

**Fig. 6:**
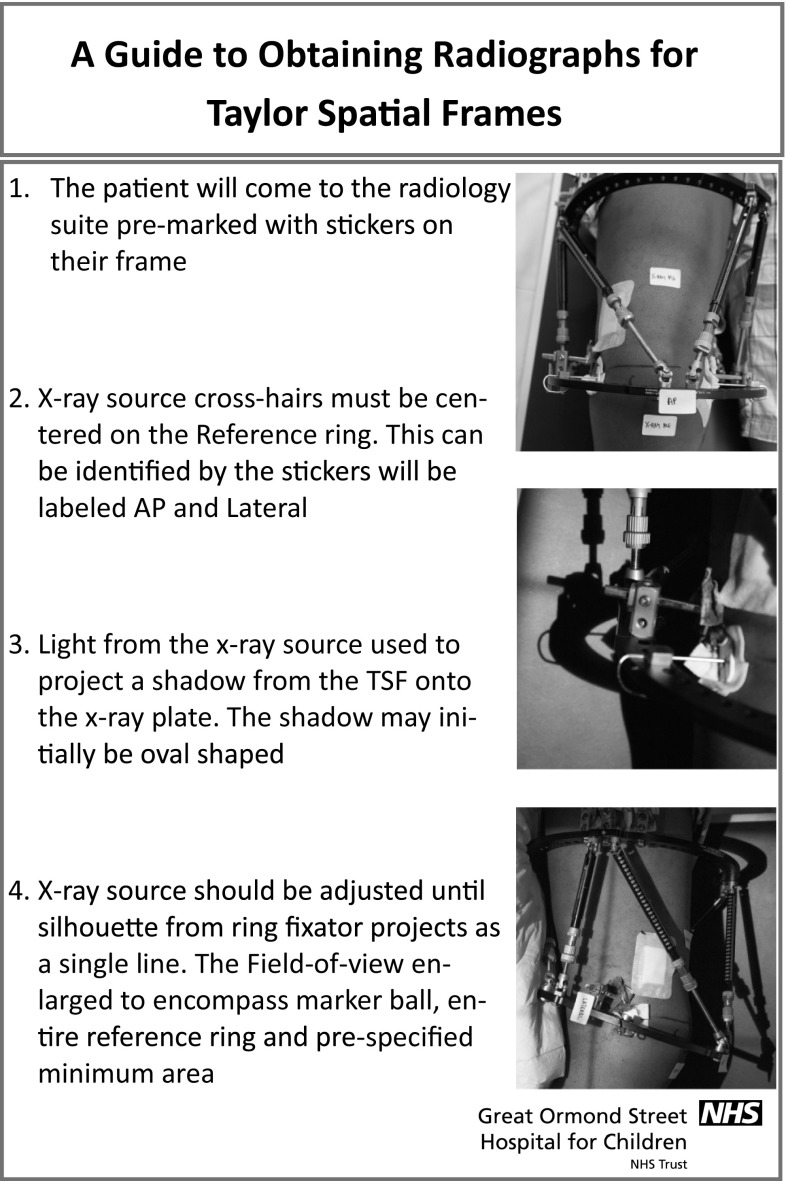
Poster used in radiology department indicating method for the silhouette technique

Following the data collection period, a poster was produced demonstrating the new technique (Fig. 6), which was displayed in each room in the main X-ray department where radiographs were undertaken. All radiographers were educated on the process by the surgical team at their weekly educational/teaching meeting to help with understanding and uptake of the method. The same text was used for the X-ray requests in both data collection periods, although in the second the additional phrase (“Please use silhouette technique”) was added.

## Results

### Outcome prior to introduction

Ten consecutive patients who underwent deformity correction were identified over a 12-month period (July 14–July 15). Median age was 11 years (range 7–18). The aetiology requiring TSF correction included Blount’s disease (2 cases), congenital short femur (2), unspecified systemic limb abnormalities (1), hypophosphataemic rickets (1), fibular hemimelia (1), hemihypertrophy (1), achondroplasia (1) and osteomyelitis (1).

The median time to first radiograph after surgery was 3 days (range 1–18 days). The median dose of radiograph was 0.25 Gy/cm^2^ (range 0.02–0.45). Only 4/10 patient’s post-operative radiographs were deemed totally adequate. The reasons identified for being inadequate were three patients whose reference ring was not orthogonal on the AP view, two patients in whom the reference ring was not orthogonal on the lateral view (including one where the a true lateral of the ring was not taken either), one patient in whom the entire ring had not been included in either AP or lateral views, and two patients in whom the longitudinal amount of bone included was not sufficient to accurately angular parameters.

### Outcome following introduction of technique

Eight consecutive patients who underwent deformity correction were identified over a 3-month period (July 2015–September 2015). The median age was 10 years (range 6–15). Aetiology included Blount’s disease (2 cases), post-sepsis growth arrest (2), post-traumatic femoral malunion (1), congenital short femur (1), hypophosphataemic rickets (1) and achondroplasia (1). The median time to first radiograph after surgery was 3 days (range 1–4 days). The median dose of radiograph was 0.18 Gy/cm^2^ (range 0.06–2.13). All patients had adequate radiographs with none requiring repeat radiographs to be taken.

## Discussion

We have described one method, which can assist in obtaining adequate post-operative radiographs for planning of correction with a hexapod external fixator (using the Taylor Spatial Frame in this example). Previously described methods have either involved construction of a custom rail in the radiography department [10] or augmentation of the frame with a spirit level device [9]. This technique was chosen, as it firstly does not require any additional equipment to be installed to either X-ray machine or the frame. Secondly, paediatric patients (the practice for this hospital) may not be able to follow commands required to position the leg as required with a spirit level.

Intra-operative imaging provides another option for achieving the same radiographic information [1, 2, 7, 8]. While this method does allow the operating surgeon to control accurately the positioning and field of view to their requirements, there are limitations in the quality of fluoroscopy imaging, and obtaining formal radiographs in theatre may add theatre/anaesthesia time. However, a recent study has demonstrated that there is no significant difference between the information obtained from either method [11].

The service evaluation demonstrated an improvement in the number of adequate post-operative radiographs obtained following introduction of the new technique to the department. There is a limitation to the interpretation of this; it is not clear to what extent the technique itself is the result of this improvement, rather than the better education of the radiographers into the requirements of a good post-op planning radiograph for a TSF. The radiographers involved did comment that it was an easy method of obtaining a good orthogonal view.

The numbers involved in the audit were small; although there was a clear improvement in the numbers of adequate radiographs (40 vs. 100%), there was minimal difference in radiation dose between the two audit cycles. One outlier for radiation dose was present in the second audit cycle although this was seen in a skeletally mature obese patient (BMI > 35 kg/m^2^) who had a similar number of X-rays taken to other patients.

Other methods described for obtaining radiographic parameters include the use of computerised tomography (CT) scanning [4]. This provides the additional benefit of allowing radiographic calculation of rotational measurements, which is usually performed through clinical measurement. Higher radiation exposure is a limitation of this technique, particularly relevant in the younger patient population. This is not a method routinely used in our institution.

## Conclusion

The silhouette sign has been demonstrated as a simple technique for assisting in obtaining adequate planning radiographs for Taylor Spatial Frame correction, without requiring additional equipment or frame modification.
